# Image-processing pipeline for diagnosing diabetic macular ischaemia in diabetic macular oedema

**DOI:** 10.1038/s41598-025-30721-y

**Published:** 2025-12-09

**Authors:** Xiaofan Xiong, Maged Habib, David H. Steel, Chingning Taffeta Yamaguchi, Tunde Peto, Boguslaw Obara

**Affiliations:** 1 School of Computing, Newcastle University, Newcastle upon Tyne, UK; 2 Biosciences Institute, Newcastle University, Newcastle upon Tyne, UK; 3 Sunderland Eye Infirmary, National Health Service, Sunderland, UK; 4 Northern Ophthalmic Research Institute, University of Sunderland, Sunderland, UK; 5 Boehringer Ingelheim, Ingelheim am Rhein, Germany; 6 Institute of Clinical Science, Queen’s University Belfast, Belfast, UK

**Keywords:** Computational biology and bioinformatics, Diseases, Health care, Medical research

## Abstract

Diabetic Macular Ischaemia (DMI) is a critical cause of vision loss, frequently occurring alongside Diabetic Macular Oedema (DMO) in patients with severe diabetic retinopathy. Accurate diagnosis of DMI is essential for assessing the visual prognosis of any intervention. In the context of clinical research for developing novel treatments to improve macular capillary perfusion, traditional imaging techniques often struggle to identify ischaemic areas precisely. Optical Coherence Tomography Angiography (OCTA) offers a non-invasive method for visualising retinal vasculature, providing potential improvements in diagnosis. However, reliable quantification of ischemia remains challenging, particularly in the presence of macular oedema, which can distort OCTA images. This study introduces an image-processing pipeline to evaluate DMI in patients with DMO using OCTA. Using a 3-dimensional methodology, the pipeline quantitatively assesses macular vascular perfusion, accounting for segmentation errors caused by macular oedema. Imaging data from 35 people with DMO and variable degrees of DMI were imaged using three OCTA devices (Heidelberg Spectralis, Optovue Angiovue, and Topcon Triton) and analysed using this 3D methodology. Key metrics including vessel density, skeletonized vessel density, average vessel radius, and our novel metric named Macular Vascular Volume (MVV), were extracted to assess consistency across two-time points, namely pre- and post-treatment with anti-Vascular Endothelial Growth Factor (anti-VEGF) agents and hence before and after resolution of macular oedema. Measurements with the novel process showed stability of the macular vessel metrics pre- and post-oedema resolution, compared with previously described OCTA metrics using current 2D segmentation methods. The proposed pipeline provides robust quantitative tools for evaluating DMI with DMO, with promise in clinical applications for improving diagnostic accuracy and optimising treatment outcomes.

## Introduction

Optical Coherence Tomography Angiography (OCTA) is a non-invasive imaging technique that employs motion contrast imaging of erythrocytes within blood vessels to capture three-dimensional details of the retinal vasculature at different depths.

OCTA has proven highly effective in diagnosing and monitoring various diseases affecting the macular, including age-related macular degeneration. It is also valuable for assessing macular vascular perfusion and ischaemia in retinal vascular conditions such as diabetic maculopathy and vascular occlusions. Quantitative analysis of 3D OCTA images of the retinal vasculature is becoming essential for standardising the objective interpretation of clinical outcomes^1^.

Several OCTA-derived features and parameters are used to quantify the retinal microvasculature. With its ability to differentiate individual vascular plexi in the retina, OCTA provides detailed insights into retinal health and pathology^2^, see Fig. 1a.

OCTA allows en-face and cross-sectional B-scan images to be generated, providing a comprehensive view of both the retinal and choroidal vasculature. En-face OCTA offers a top-down view of vascular layers, while cross-sectional B-scans also include structural information, aiding in the localisation of the exact depth of vascular abnormalities. Several layers of capillary plexi are described, but by convention, these are divided into the superficial vascular plexus (SVP), within the inner retina and the deep vascular complex (DVC) comprising the deep and intermediate capillary plexi in the deeper layers. The accurate extraction and estimation of OCTA-derived metrics rely mainly on the precise segmentation of the retinal layers to identify these vascular complexes^3^, see Fig. 1b. However, misidentification of the different retinal layers or segmentation errors leads to inaccurate measurements and incorrect diagnoses. The presence of retinal pathology that distorts normal retinal shape, such as macular oedema with fluid cysts, scarring, or the masking effect of macular haemorrhages or exudates, can also contribute to OCTA segmentation errors requiring manual correction for reliable results^2^.

The project aimed to develop an image-processing pipeline to accurately quantify macular vascular perfusion metrics despite the presence of Diabetic Macular Oedema (DMO). This is a key step towards identifying the presence and degree of any Diabetic Macular Ischaemia (DMI) present. DMI is a major cause of visual loss in diabetic patients and is associated with decreased macular vessel density and/or non-perfusion of the vascular plexi. We evaluated the robustness of this novel technique by applying it to OCTA images collected in patients with DMO, before and after treatment with anti-vascular endothelial growth factor (anti-VEGF) agents. We compared the performance of the novel 3D pipeline in terms of the consistency of the vascular metrics before and after oedema resolution, as compared to existing 2D techniques and in control eyes without macular oedema.

## Related work

### Clinical applications

Several OCTA metrics have been used in clinical practice and trials of DMO and DMI. The Foveal Avascular Zone (FAZ) is the region at the foveal centre that is free of retinal capillaries. It typically has a diameter of approximately 500 micrometres. Loss or non-perfusion of the capillaries at the FAZ margin, along with enlargement of the FAZ area, is negatively associated with visual acuity. However, the correlation between FAZ area and Best Corrected Visual Acuity (BCVA) is poor due to the significant physiological inter-person variability in FAZ size^4^. This variability tends to limit the FAZ’s utility in confirming macular ischaemia unless baseline images are obtained before the onset of pathology^5^. Longitudinal data are hence more meaningful than cross-sectional data.

Additionally, the concurrent presence of other pathologies, such as epiretinal membrane and/or macular oedema, can affect the size and contour of the FAZ by altering the retinal surface shape with consequent complex layer segmentation and possible faulty vessel discrimination in the individual retinal plexi. Treatment of these conditions can lead to changes in vessel positions, resulting in artefactual changes in FAZ estimation.

Another important and useful metric for estimating vascular perfusion in the macular area is vessel density. This quantitative measure approximates the number of vessels in a specific retinal region. Vessel density can be assessed as the proportion of the area with blood flow relative to the total measured area (vessel area density) or as the total length of perfused vessels within an area of interest (skeletonized vessel density)^6^. Skeletonized vessel density is preferred because it is less affected by image resolution and apparent vessel calibre^7^. However, non-skeletonized methods may provide more comprehensive information about the vascular network since they account for vessel size^8^. Accurately identifying vessel density requires reliable algorithms that address projection artifacts, shadow artifacts, and motion artifacts. Precise estimation of vessel density hinges on accurate segmentation of the individual capillary plexuses, which can be particularly challenging in the presence of structural abnormalities associated with diabetic retinopathy or macular oedema. Indeed, misidentification of retinal layers or segmentation errors is a significant source of artifacts in 33–100% of OCTA images and can occur with any OCTA device^9^. These errors can lead to substantial alterations in extracted OCTA parameters, potentially resulting in incorrect diagnoses. Therefore, it has been proposed to evaluate the entire retinal thickness, providing data for all retinal layers^4^.

### The effect of anti-VEGF on retinal vasculature

The effect of anti-VEGF treatment, used routinely to treat DMO, on macular capillary perfusion has been debated, with varied interpretations. Resch et al.^10^ reported reduced vessel density in both the SCP and DCP following one year of treatment with anti-VEGF injections^10^. This finding was not supported by Lee et al., who reported vessel density to be more affected by age than anti-VEGF injections^11^. Although other authors have reported changes in retinal perfusion over long periods of anti-VEGF, such changes have not been reported after short-term or limited number of anti-VEGF injections course as applicable for this study^1,12^. For studies focusing on short-term measurements, Cennamo et al.^13^ found no significant differences in vessel density following short-term treatment as compared with baseline. Arumuganathan et al.^14^ reported the stability of OCTA measurements after 1 day, 1 week and 1 month following anti-VEGF treatment.

For this project, we assumed that macular perfusion was stable over time, and any detectable inconsistencies between microvascular measurements from the two OCTA scans pre- and post-short-term treatment with anti-VEGF were attributable to estimation errors due to the presence of DMO rather than changes in the retinal vasculature. Capillary perfusion parameters were estimated with the 3D algorithm at a global retinal level without segmentation into the DVC and SCP. For comparison with 2D techniques, we focused on the DVC measurements as this plexus has been reported as being more affected than the SCP in diabetic retinopathy and more sensitive to disease progression and response to treatments^13,15^.

### Image processing approaches

Most existing methods for processing OCTA images rely on 2D en-face OCTA maps (C-scans) derived from volumetric OCTA datasets of any retinal layer of interest. These maps are typically generated through Maximum Intensity Projection (MIP). There are two primary reasons for focusing on 2D projections rather than the original 3D volumes: (1) MIP helps to ignore projection artifacts, resulting in a cleaner angiogram, and (2) using 2D images facilitates direct comparison with other 2D techniques, such as Fluorescein Angiography (FA), by applying established quantitative metrics like blood vessel density, skeleton vessel density, and FAZ area. However, creating 2D projections of the 3D volumetric vascular network can be demanding, particularly when imaging diseased retinas. For instance, intra-retinal fluid accumulation caused by DMO can disrupt macular structures and complicate the automated segmentation of retinal layers, leading to inaccurate projection images. Additionally, the inherent information loss in 2D projections can result in altered metrics, such as branch points where vessels appear to overlap in 2D but are actually at different elevations in 3D. Therefore, a full-volume analysis method for 3D OCTA is highly desirable^16^.

Recent deep-learning algorithms have shown promising results for vascular segmentation in 2D^17^. However, 3D retinal vessel segmentation in OCTA remains challenging due to the scarcity of high-quality 3D manual annotations, which are challenging and time-consuming to obtain. As an alternative approach, we attempted to train 2D segmentation networks using the public ROSE^18^ dataset and then applied these trained networks to 3D volumes in a slice-by-slice manner. The results were suboptimal because the ROSE dataset contains only MIP images, and our experiments revealed that networks trained on MIP images do not generalise well to slices from 3D volumes (unpublished data).

Among the few available methods for 3D OCTA vascular segmentation, Zhang et al.^19^ proposed using the Optimally Oriented Flux (OOF)^20^, which produced the most visually promising results. However, no quantitative analysis was conducted due to the lack of ground truth 3D segmentation. Consequently, we have chosen to utilise the OOF as our segmentation algorithm.

## Materials

**Fig. 1:**
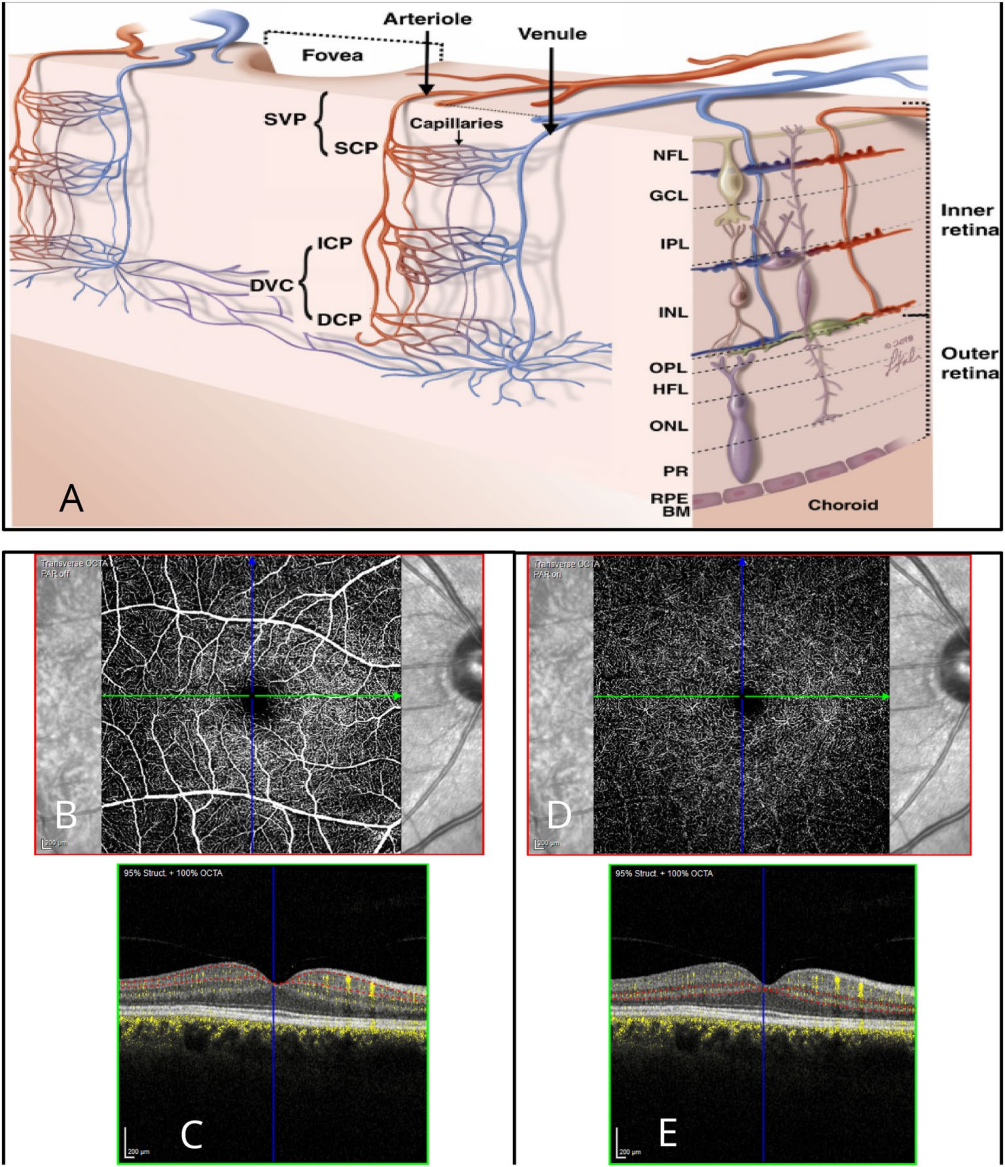
(**a**) Showing a schematic representation of the retinal vascular plexi in the peri-foveal area. Panels (**b**) and (**d**) are the en-face slab view of the SVP (superior vascular plexus) and DVC (Deep vascular complex). Panels (**c**) and (**e**) are the central horizontal flow B scans for the corresponding vascular complexes. $*$ DVC comprising the ICP and DCP (intermediate and deep capillary plexus). Taken from:^21^. NFL, Nerve fibre layer; GCL, ganglion cell layer; IPL, inner plexiform layer; INL, inner nuclear layer; OPL, outer plexiform layer; HFL, Henle’s fibre layer; ONL, outer nuclear layer; PR, photoreceptor outer segments; RPE, retinal pigment epithelium; BM, Bruch’s membrane.

### Medical protocols

OCTA manufacturers apply differing algorithms to measure the retinal vasculature, and we, therefore, sought to compare our developed system to several devices. OCTA scans from three different manufacturers’ devices (Heidelberg Spectralis (Heidelberg Engineering GmbH., Heidelberg, Germany), Optovue Angiovue (Optovue Inc. California, United States), and Topcon Triton (Topcon Healthcare. Tokyo, Japan)) were captured from diabetic patients with active DMO undergoing treatment with Aflibercept as part of a prospective clinical trial: *Image Analysis Approach for Quantitative Evaluation of Diabetic Macular Ischemia in the Presence of Macular Oedema on OCTA Images. The study received the ethical approval from the comprehensive local research network CLRN (The DIME Study, NIHR CPMS ID 48908)*. The study was strictly conducted in accordance with regulations under the declaration of Helsinki. All patients signed an informed consent before recruitment. The order of image capture by devices was varied cyclically between patients to avoid bias to any particular device.

Forty-four patients were recruited for the study. Nine patients were excluded as withdrawn or deceased before the end of the study visit. Thirty-five eyes with DMO (designated as ‘study eyes’) from 35 patients were evaluated as part of the study. The mean age was 56.8 years old, 26 (74%) were male, 19 were right eyes, and 23 (65%) were treatment-naïve. 13 (37%) had type 1 and 22 (63%) type 2 diabetes. OCTA metrics were extracted using the new 3D processing pipeline to describe the vascular network in these patients, and the consistency of the metrics between the two visits (baseline and end of study, 3 to 5 months later after 3–5 doses of Aflibercept irrespective of the treatment outcome) was assessed. The consistency of the metrics in the study eyes was compared to 23 non-treated fellow eyes without DMO to ascertain the effect of oedema on the variability of the resulting outcomes. As for the study eyes, the baseline mean central macular thickness (CMT) (in the presence of oedema) was 483 microns, this improved to 310 microns at the end of the study visit (with a resolution of oedema) with the Heidelberg Spectralis OCT machine. 32 out of the 35 study eyes (91%) achieved a dry macula after the treatment course.

The results from the 3D pipeline were also compared to those obtained from current techniques utilising 2D image scan segmentation. The 2D FAZ estimates were calculated by an accredited retinal image reading centre (Belfast Ophthalmic Reading Centre, Centre for Public Health, Queen’s University, Belfast, UK).

### OCTA data collection

The following imaging protocols were utilised for the three OCTA devices used:

- Optovue Angiovue: 6 $\times$ 6 mm scan area (each B scan 400 $\times$ 400 pixels with 15 microns spacing between scans, 400 B scans in total per volume scan).
- Topcon Triton: 6 $\times$ 6 mm (320 $\times$ 320 pixels with 18.7 microns spacing between scans, 320 B scans in total per volume scan).
- Heidelberg Spectralis: 5.4 $\times$ 5.4 mm (512 $\times$ 512 pixels with 10.63 microns spacing between scans, 510 B scans in total per volume scan).

Images from each machine can be either raw or processed using the respective in-machine algorithms, where image contrast enhancements, eye motion artifact removal, and denoising take place. To ensure a fair comparison, we focused exclusively on raw images from all three machines. This approach avoided potential biases that could be introduced by the different in-house processing algorithms used by each manufacturer.

### OCTA data quality assessment

**Fig. 2:**
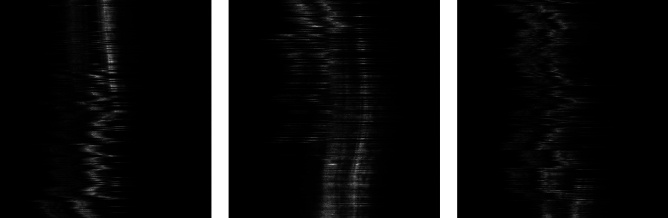
Examples of YZ projections of low-quality 3D OCT images from Heidelberg.

**Fig. 3:**
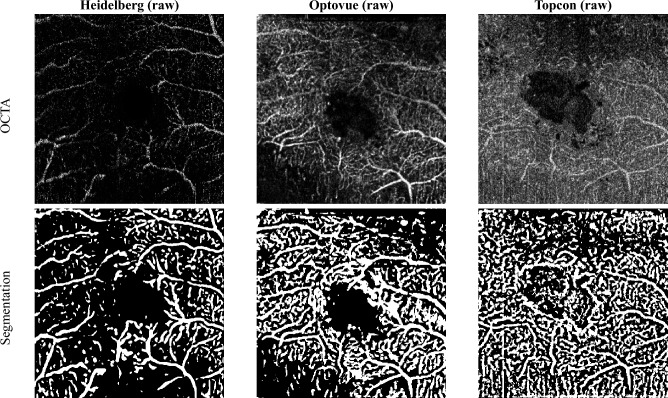
Examples of raw OCTA images (2D slices) of the same patient eye at the same region, from the three machines (Heidelberg, Optovue, and Topcon) and corresponding binary segmentation results.

Another important factor considered was the image quality. In general, the images from the three machines had comparable qualities (samples shown in Fig. 3. However, some images (20 out of 73 (27.4%)) from Heidelberg had significant artifacts that could be best seen in maximum projection images. This issue was not observed in images captured by other manufacturers and might have occurred due to patients’ compliance with increased eye movements during the longer image capture procedure compared to the other 2 machines^22^ (Fig. 2).

## Methods

### 2D OCTA scan analysis

The OCTA scans were captured by the three devices; Optovue AngioVue, Heidelberg Spectralis and Topcon Triton using a standardised protocol. They were extracted, anonymised and transferred to be graded by the Belfast Ophthalmic Reading Centre, Queen’s University, Belfast, UK. For the Optovue AngioVue and the Topcon Triton, the device-generated FAZ estimated areas were reviewed and recorded. This was for the whole retinal thickness encompassing all vascular complexes for Optovue, and separately for the DVC for the Topcon Triton device. Manual adjustments and corrections were performed where deemed necessary. For the Heidelberg Spectralis machine, manual plotting of the FAZ area was undertaken with the image annotation tool within the Heidelberg platform in the DVC machine-segmented layer. All recorded area measurements were checked and tabulated by trained and certified image graders with adjudication of 10% of the data for quality assurance as per the established centre protocol. All graders were masked regarding the relevant clinical information.

**Fig. 4:**
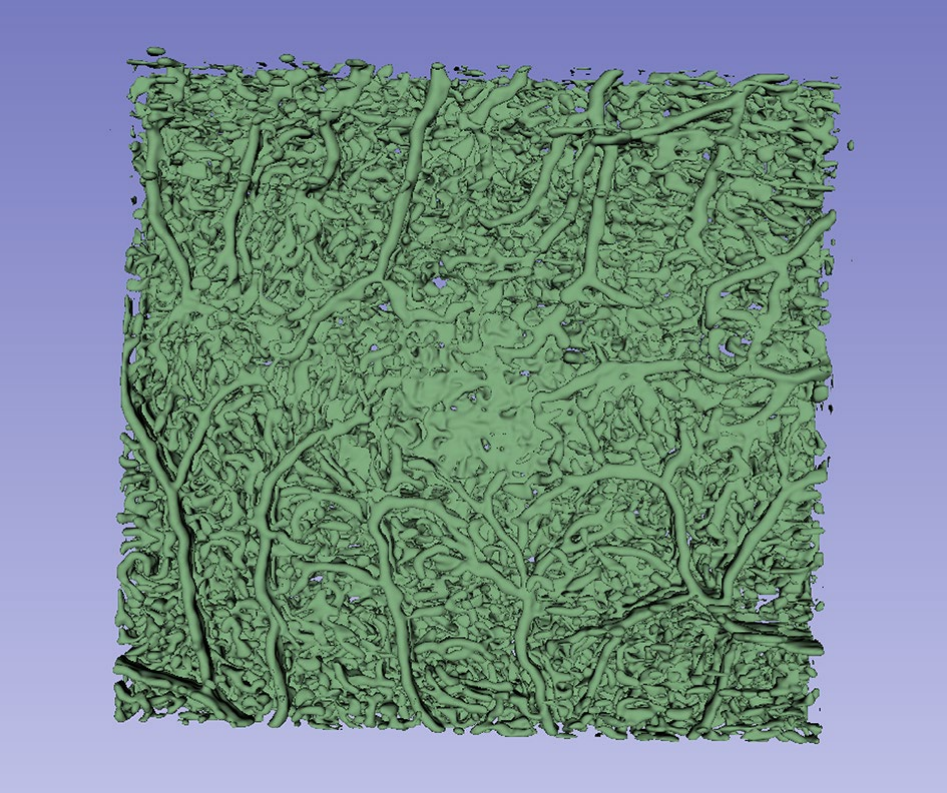
A 3D rendering of a typical segmentation mask.

### 3D OCTA scan processing

The image processing pipeline contained two main steps, described as follows.

1. Creating 3D volume of interest (VOI): Original raw image files from Optovue machines were 2D images with various sizes and fields of view (FOV) that contained one or both eyes. Therefore, all images for each patient were first stacked to create a 3D volume and then split to isolate individual eyes. Raw images from Heidelberg machines were provided as 3D volumes in the “.vol” format per eye, which were then converted to TIF files for easier processing. Raw images from Topcon were provided as DICOM files for each eye and were also converted to TIF.
2. Vessel segmentation: As mentioned in section, due to the absence of 3D manual annotations, we found that the OOF was the optimum method for retinal vessel segmentation in 3D OCTA images. Thus, OOF was applied to the processed images from the previous step to obtain 3D vessel segmentations.

**Fig. 5:**
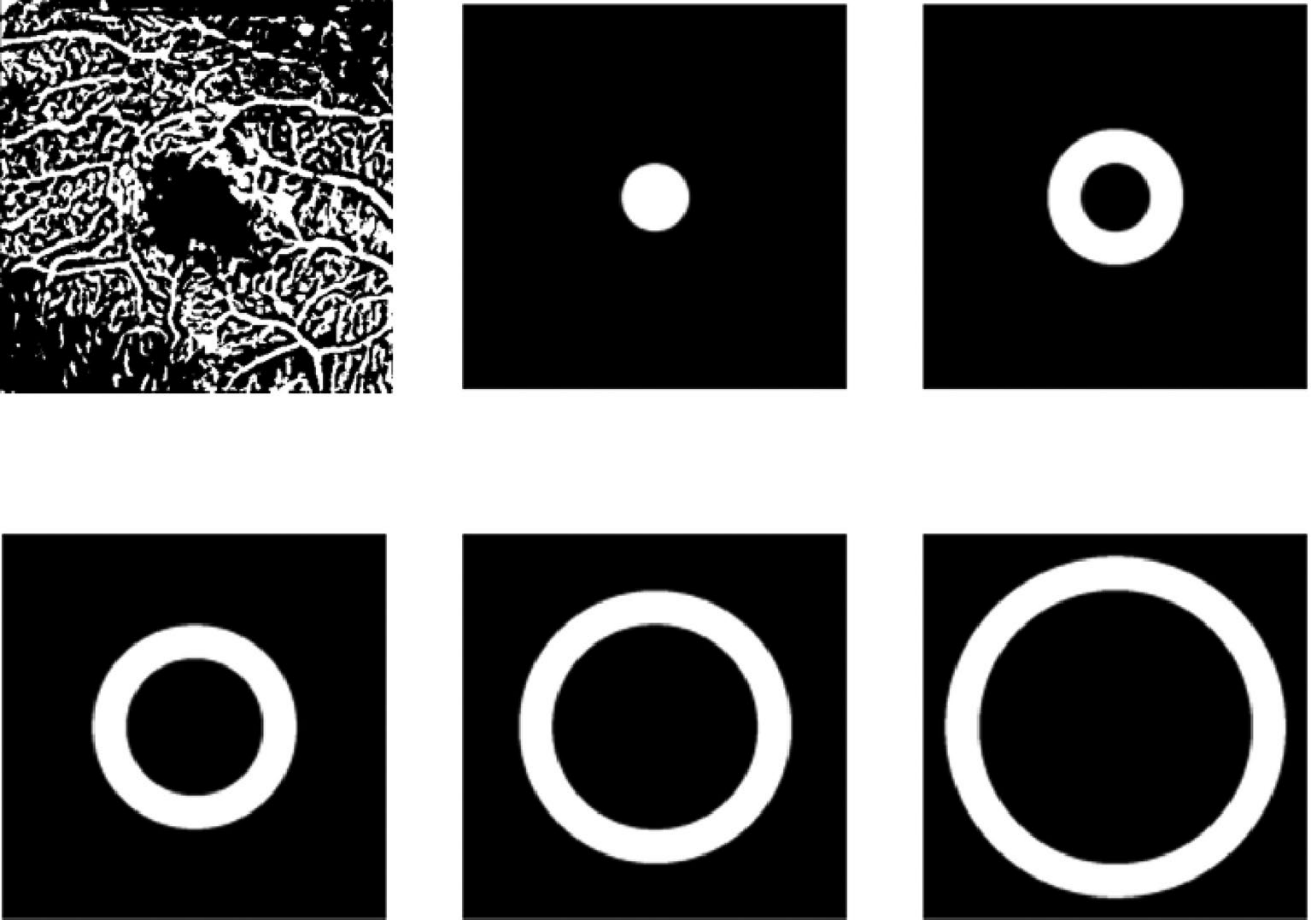
We define concentric cylindrical tubes for segmented OCTA images, starting with the centre of mass, which defines the centre of the 3D cylinder, and then design tubes defined by incrementally larger radii.

### 3D OCTA scan analysis

We studied five quantitative metrics derived from the 3D vessel segmentation:

1. Blood vessel density: The percentage of vessel volume (voxels) in the image.
  1 $$\begin{aligned} \textit{blood vessel density} = \frac{\sum {V_{v}}}{\sum {V_{i}}} , \end{aligned}$$
  where $V_{v}$ represents the segmented vessel voxels, and $V_{i}$ represents all voxels in the OCTA image.
2. Skeleton vessel density: The percentage of vessel skeleton volume (voxels) in the image. The vessel skeleton was generated by thinning the segmented vessels to a 1-voxel width.
  2 $$\begin{aligned} \textit{skeleton vessel density} = \frac{\sum {V_{s}}}{\sum {V_{i}}} , \end{aligned}$$
  where $V_{s}$ represents the skeleton voxels, and $V_{i}$ represents all voxels in the OCTA image.
3. Average vessel radius: The average radius of vessels in units of the voxels. This was calculated by performing a distance transform on the vessel skeleton, where each voxel on the vessel surface has a corresponding distance to the nearest skeleton, representing the radius.
  3 $$\begin{aligned} \textit{average vessel radius} = \frac{\sum {D_{cs}}}{\sum {V_{c}}} , \end{aligned}$$
  where $D_{cs}$ is the distance from each vessel surface voxel to the nearest skeleton, and $V_{c}$ represents all voxels on the segmented vessel surface.
4. Total surface volume: The total number of voxels on the vessel surface.
  4 $$\begin{aligned} \textit{total surface volume} =\sum {V_{c}} , \end{aligned}$$
  where $V_{c}$ represents all voxels on the segmented vessel surface.
5. Macular vascular volume (MVV): Defining the FAZ in a 3D volume is challenging, so we propose an alternative novel metric named Macular Vascular Volume (MVV) that focus on measuring the total macular vascular volume across the whole scan. This metric calculates the vessel volume within concentric cylindrical tubes with incrementally increasing radii. As shown in Fig. 5, five cylindrical tubes were created for each OCTA image. The total volume of vessels inside each cylinder was calculated by applying the cylinder as a binary mask to the vessel segmentation and summing the vessel voxels inside the cylinder.

**Fig. 6:**
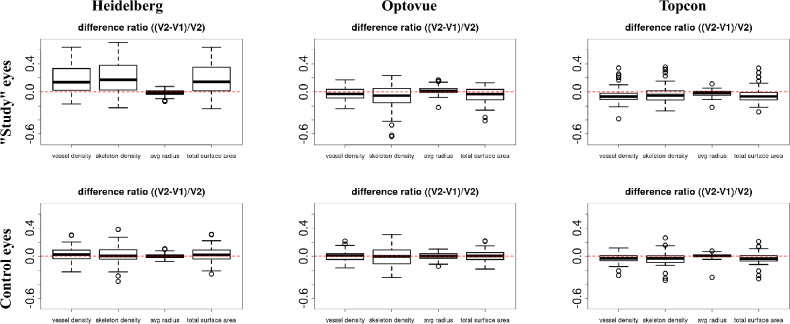
Box plots of the *difference ratios* of four metrics between before (*V*1) and after (*V*2) the treatment on “study” eyes (First row) and control (healthy) eyes (second row).

### Segmentation visualization

Due to the lack of reference annotations, a quantitative analysis of the segmentation results is not possible. Therefore, we present the visualisation of typical segmentation results in Fig. 3 and a 3D rendering of a segmentation mask in Fig. 4.

**Fig. 7:**
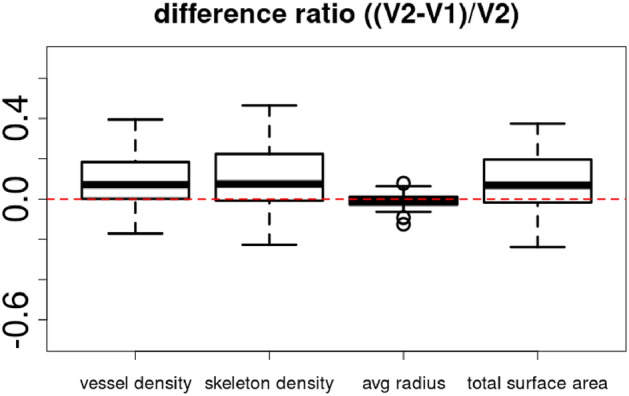
Metrics of Heidelberg device, excluding low-quality images.

### Quantitative analysis

For raw images of “study” and “control” eyes from the three machines, the difference ratio between pre-treatment (*V*1) and post-treatment (*V*2) for each quantitative metric described in Section 1.9 was calculated using the equation:

5 $$\begin{aligned} \textit{difference ratio} = \frac{V2-V1}{V2}. \end{aligned}$$

**Fig. 8:**
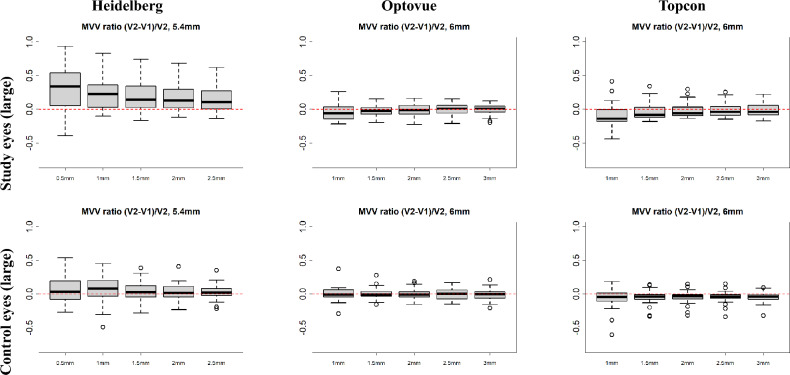
Comparison of MVV between pre- (V1) and post- (V2) treatment images from the three machines. The difference ratios ($(V2-V1)/V2$) of vessel volumes inside the five cylinders are shown for study (first row) and control eyes (fourth row). The X-axes show the radius of each of the five cylinders, while the Y-axes show the difference ratios.

## Results

The boxplots of the difference ratios of the three devices for the quantitative metrics 1–4 are shown in Fig. 6. Notably, all difference ratios (DiffR) are significantly different from 0, based on the t-test results. Also, as “OCTA data quality assessment” mentioned, a subset of Heidelberg images had severe artifacts. Therefore, a boxplot with low-quality images excluded is shown in Fig. 7.

For the MVV, the difference ratios for all cylinder cubes are shown in Fig. 8. The vessel volume in each cylinder is normalised by the volume of the cylinders to avoid bias caused by different cylinder sizes. One observation is that the further away the cylinder is from the centre, the smaller the difference ratio.

In the study’s eyes, the mean of the DiffR for the MVV were − 0.02, 0.21, and − 0.03 for the Optovue, Heidelberg, and Topcon machines, respectively. In comparison, the mean DiffR for 2D DVC FAZ areas was higher at − 0.08, − 1.24, and − 0.59 for corresponding devices. The 3D mean DiffR were significantly lower than 2D DiffR for the study eyes for the Heidelberg and Topcon devices, but not the Optovue. The variance was notably lower with the 3D method for all devices. See Table 1.

For the control eyes, the mean DiffR for the MVV were comparable to the study DMO eyes, namely − 0.001, 0.03, and − 0.05 for the Optovue, Heidelberg, and Topcon machines respectively, although the variance for the Heidelberg machine was notably higher for vessel and skeleton density and total surface area in the study eyes. The mean DiffR for 2D DVC FAZ areas were 0.04, − 0.01, and − 0.68 for the corresponding machines, respectively. There was no statistically significant difference between 3D and 2D DiffR for control eyes for the Optovue and Heidelberg devices, but there was a significant difference with the Topcon. Again, the variance with the 3D method was notably lower with all three devices. See Table 1.

**Table 1:** Summary of difference ratios between 2 visits using 3D and 2D methodologies for the three devices. Numbers in each entry are in the format of “mean (standard deviation)”.

	Study eye			Control eye		
	3D MVV	2D FAZ area	p-value	3D MVV	2D FAZ area	p-value
Optovue angiovue	− 0.02 (0.02)	− 0.08 (0.54)	0.514	− 0.001 (0.004)	0.04 (0.34)	0.478
Heidelberg spectralis	0.21 (0.06)	v1.24 (1.29)	< 0.001	0.03 (0.01)	− 0.01 (0.31)	0.448
Topcon triton	− 0.03 (0.03)	− 0.59 (0.72)	< 0.001	− 0.05 (0.01)	− 0.68 (0.84)	< 0.001

## Discussion and future work

In this study, we aimed to address the challenges and problems of analysing macular capillary perfusion with 2D OCTA scans in the presence of retinal thickening and oedema as a result of poor retinal layer segmentation. We propose a first attempt at a full-volume analysis method for 3D OCTA, aiming to quantitatively compare macular perfusion before and after DMO treatment, overcoming segmentation defects to achieve consistent quantitative assessments independent of DMO distractors. Our results demonstrated consistent measurements and estimates of all the tested parameters between the two study visits before and after treatment in the presence and absence of macular thickening. Our results demonstrated more consistent MVV measurements with the 3D pipeline as compared to conventional 2D segmentation methods. The difference reached statistical significance in the study eyes with the Topcon and Heidelberg devices, highlighting the impact of macular oedema on automated layer segmentation and resultant disparities in FAZ estimation. The differences between the 3D and 2D methodologies were less pronounced in the control eyes with no macular oedema, although again, the variance in the difference ratios was markedly lower with the novel 3D methodology. The resultant overall consistency in the examined parameters with the novel 3D metrics might be considered clinically acceptable, especially when compared with the existing 2D methodologies as well as in comparison with the previous study exploring short and long-term repeatability of OCTA parameters in healthy subjects^23^. The main advantage of the proposed method is that, unlike current 2D methods that rely on accurate retinal layer segmentation to create MIP, 3D segmentation appears to be less prone to the negative impact caused by the presence of intra-retinal and sub-retinal fluid.

We also acknowledge that the study has several limitations, the most significant being the lack of reference data, which complicates the quantification of segmentation performance and the development of supervised or semi-supervised deep learning-based approaches. Given the impracticality of creating manual annotations for the entire 3D OCTA dataset, exploring synthetic data may be a valuable alternative. Furthermore, our current processing pipeline does not include a retinal layer segmentation step to create a more refined VOI that consists of all retinal vessels. This omission is due to the lack of reference 3D annotations of the internal limiting membrane (ILM) and Bruch’s membrane (BM) in similar diseased conditions, and needs to be studied in the future.

Further work is thus required to validate the measurements obtained by the proposed 3D analysis methods. Currently the radius selected for the MVV focus on validating the metrics on vessels across the whole scan, which has proved to be consistent. Future directions include a more refined study on MVV using smaller cylinder radius, making the metrics focusing on a smaller region comparable to the area of traditional 2D FAZ. We also plan to explore the repeatability of our findings with other OCTA imaging protocols and scan sizes, as well as other macular capillary perfusion parameters. The reliability of the 3D methodology also needs to be studied in terms of the diagnosis of DMI based on current definitions.

## Data Availability

The imaging data is not available for open access based on local information governance policies, however it can be accessed on reasonable request from the authors with the completion of suitable data sharing agreements.
